# Anesthesia management for thoracoscopic resection of a huge intrathoracic meningocele: a case report

**DOI:** 10.1186/s40981-024-00697-1

**Published:** 2024-02-19

**Authors:** Ryosuke Nakazawa, Kenichi Masui, Takahisa Goto

**Affiliations:** Department of Anesthesiology, Yokohama City University School of Medicine, 3-9 Fukuura, Kanazawa-ku, Yokohama, 236-0004 Japan

**Keywords:** Anesthesia management, Huge intrathoracic meningocele, Neurofibromatosis type 1, Cerebrospinal fluid pressure monitoring

## Abstract

**Background:**

Diagnosed intrathoracic meningocele is an uncommon complication of neurofibromatosis type 1. We report an anesthesia management for a rare case undergoing thoracoscopic resection of a huge intrathoracic meningocele.

**Case presentation:**

A 51-year-old woman was scheduled for thoracoscopic meningectomy under general anesthesia. We monitored intrathecal pressure during anesthesia to prevent a decrease in intrathecal pressure. During surgery, the intrathecal pressure occasionally increased by around 5 cmH_2_O immediately after the insertion of the drainage tube and occasionally decreased by up to 10 cmH_2_O during the careful slow aspiration of the cerebrospinal fluid (CSF). The pressure rapidly recovered after the interruption of the procedures. She was discharged on postoperative day 4 without major complications.

**Conclusions:**

The CSF pressure was fluctuated by procedures during thoracoscopic resection of a huge meningocele. A CSF pressure monitoring was useful to detect the sudden change of CSF pressure immediately, which can cause intracranial hemorrhage.

## Background

Diagnosed intrathoracic meningocele is an uncommon complication of neurofibromatosis type 1 [1] although more patients might have small intrathoracic meningoceles without symptoms. Huge meningocele can interfere with lung expansion and can cause respiratory distress. As no case report of anesthesia management for intrathoracic meningocele surgeries was found, we report a case undergoing thoracoscopic resection of a huge intrathoracic meningocele which occupied larger than one-third of the volume of the left thoracic cavity.

## Case presentation

**Fig. 1:**
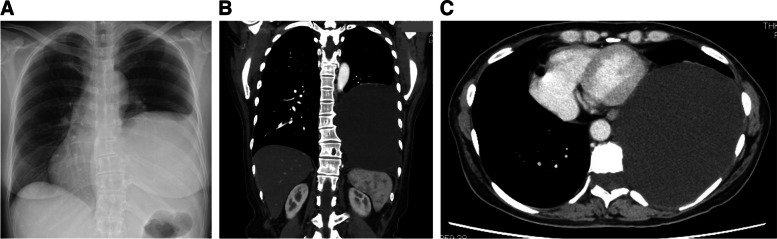
Preoperative chest X-ray image (**a**), and preoperative coronal (**b**) and axial (**c**) computed tomographic views showing the huge intrathoracic meningocele

Written patient consent was obtained for the report. A 51-year-old woman had been pointed out with a pulmonary cyst on medical checkup since childhood. After a recently detailed examination due to respiratory distress, she was diagnosed to have a large intrathoracic meningocele as a complication of neurofibromatosis type 1. The meningocele (130×146×150 mm) occupied larger than one-third of the left thoracic cavity and compressed the mediastinum (Fig. 1). The meningocele was connected to the subarachnoid cavity at the 9th thoracic vertebra level. She was scheduled for a thoracoscopic meningectomy. Before the surgery, the spirometry test showed restrictive ventilatory impairment (volume capacity: 1.92 L, %volume capacity: 66.4%, forced expiratory volume 1: 1.68 L, forced expiratory volume 1%: 85.7%) and her peripheral oxygen saturation was 98% at room air. The surgical procedure could significantly alter intrathecal pressure during surgery and could cause cerebrospinal fluid leakage after surgery. A sudden or persistent decrease in intrathecal pressure can cause intracranial hemorrhage. Therefore, an intrathecal pressure monitoring was planned during and after the surgery using a cerebrospinal fluid (CSF) drainage tube.

Anesthesia was induced with propofol 90 mg, remifentanil 0.2 μg/kg/min, and rocuronium 40 mg followed by tracheal intubation using a 35-Fr double-lumen tube and was maintained with sevoflurane 1.5–2%, fentanyl (total dose of 300 μg), and remifentanil 0.1–0.2 μg/kg/min. After the anesthesia induction, she was positioned in the right lateral position and right lung ventilation was performed. A standard CSF drainage tube was inserted approximately 5 cm into the intrathecal space between the 3rd and 4th thoracic vertebra to monitor intrathecal pressure continuously. Heart rate and noninvasive arterial blood pressure were stable throughout the surgery, while the intrathecal pressure was increased and decreased by the surgical procedures as follows. In the early stage of the thoracoscopic procedure, the intrathecal pressure occasionally increased by around 5 cmH_2_O compared to the control level immediately after the insertion of the drain due to the compression of the thoracic meningocele. Then, a thick intravenous plastic catheter was inserted into the meningocele to aspirate the CSF using a 50-mL syringe. During the careful slow aspiration of the CSF (average rate was approximately 50 mL/min), the intrathecal pressure occasionally decreased by up to 10 cmH_2_O. When the aspiration was interrupted, the intrathecal pressure rapidly recovered to the baseline. We informed the surgeons of a large pressure difference compared to the baseline and of a rapid pressure change, if necessary. A total of 2000 mL CSF was drained in 40 min. After opening the thoracic meningocele and confirming the connection between the lumen of the meningocele and intrathecal space, the meningocele was resected and the resection site was tightly sutured. Then, the intrathecal pressure became stable at the control level. As postoperative intrathecal pressure was expected to stabilize due to the tight sutures, we determined that postoperative intrathecal pressure monitoring was unnecessary. Accordingly, the CSF drainage tube was removed immediately after the completion of the surgery. She recovered from general anesthesia without remarkable complications, and no intracranial hemorrhage was found on the examination of computed tomography immediately after surgery.

Postoperatively, the patient had a mild headache in the sitting position, which may have been caused by CSF hypovolemia. This headache disappeared on postoperative day 3 without treatments. No major complications including neurological deficits were found until the patient was discharged on postoperative day 4.

## Discussion

Intrathoracic meningoceles are uncommon anomalies of which 70% are associated with neurofibromatosis type 1 [2]. While small meningoceles generally cause no symptoms, a huge intrathoracic meningoceles can result in patient discomfort such as respiratory distress. To reduce the size of intrathoracic meningoceles, a cyst-peritoneal shunt is one choice of the first surgery because the surgical treatment is difficult due to its fairly uncommon pathology [1]. However, the shunt may result in limited or no improvement of the symptoms. Other surgical treatments were reported including resection or plication of the meningocele [1, 3]. In the present case, thoracoscopic resection was selected as the surgical treatment.

CSF monitoring was implemented using a CSF drain tube in the present case. In the early stage of the surgery, the intrathecal pressure was occasionally elevated by around 5 cmH_2_O during mild compression of the huge meningocele to secure the surgical field. As the surgical field was narrow because of the huge meningocele and thoracoscopic procedure, the elevation of CSF pressure was unavoidable. The CSF pressure monitoring was likely to prevent longer and higher cerebrospinal pressure which might cause lower cerebral perfusion or neurological deficit. During the aspiration of the CSF in the meningocele, intrathecal pressure was occasionally decreased. As a rapid aspiration of CSF with a decrease in CSF pressure is similar to excessive CSF drainage, the aspiration can result in intracranial bleeding [4]. In patients with CSF drainage undergoing surgeries for thoracoabdominal aortic aneurysms, 10% of patients had bloody CSF and half of these had intracranial hemorrhage when intrathecal pressure maintained lower than 8 mmHg (= 10.9 cmH_2_O) [5]. In the present case, the measured pressure occasionally decreased by up to 10 cmH_2_O compared to the control level during CSF aspiration. The cumulative duration of the pressure reduction was short and the rapid aspiration was avoided with monitoring may help to prevent intracranial bleeding which was confirmed immediately after the surgery. Without CSF monitoring, it would be difficult to assess such CSF pressure changes. The intraoperative CSF monitoring was likely to be useful.

The CSF drainage tube was not used for postoperative care in the present case. In previous case reports, a CSF drainage tube was also used postoperatively for a week to maintain cerebrospinal pressure at a normal level [6, 7] to prevent CSF leakage via the surgical site. In the present case, the tube was removed because tight suture was visually confirmed. Valsalva maneuvers will be useful for robust confirmation of the absence of CSF leakage although we did not perform that [1]. In the present case, mild headache for 3 days but not major complications were observed postoperatively.

Although the intraoperative insertion of a CSF drainage tube has been reported to be useful, the procedure is associated with complications. Complications include post-dural puncture headache, intracranial hemorrhage, meningitis, and epidural abscess, and it was reported that 12.7% of patients had some kind of complication from the procedure [8]. It has also been reported that one-third of spinal cord injuries that occurred during the perioperative period of aortic surgery were caused by the insertion of a CSF drainage tube [9]. We should realize these risks to determine whether a CSF drainage tube is applied.

We experienced anesthesia management for thoracoscopic resection in a patient with a huge intrathoracic meningocele. CSF pressure monitoring during surgery was useful to detect the cerebrospinal pressure change to prevent intracranial bleeding due to excessive reduction of CSF pressure during aspiration of CSF in the meningocele. If there is a possibility of CSF leakage via the resection site of meningocele postoperatively, CSF monitoring may help to find major leakage.

## Data Availability

Not applicable.

## Abbreviation

CSF: Cerebrospinal fluid
